# Editorial: Editor’s challenge: Dr. Sara Pilotto - modulating tumor-host interplay through lifestyle in lung cancer

**DOI:** 10.3389/fonc.2026.1776689

**Published:** 2026-01-15

**Authors:** Alice Avancini, Morten Quist, Sara Pilotto

**Affiliations:** 1 Department of Neurosciences, Biomedicine and Movement, University of Verona, Verona, Italy; 2 Section of Innovation Biomedicine -Oncology Area, Department of Engineering for Innovation Medicine (DIMI), University of Verona and University and Hospital Trust (AOUI) of Verona, Verona, Italy; 3 Rigshospitalet, University Hospital of Copenhagen, Copenhagen, Denmark

**Keywords:** body composition, cancer, exercise, immune system, lung cancer, mechanisms

Lung cancer remains the most common malignancy worldwide, but its care is undergoing a profound revolution. Previously conceptualized as aberrant cellular proliferation, it is now increasingly understood as a systemic and dynamic disease shaped by interactions among the tumor microenvironment, host factors, treatment, and lifestyle features. Lifestyle interventions, and exercise in particular, are moving from the sole concept of supportive care to a biologically meaningful adjunctive strategy capable of influencing tumor-host dynamics. Clinicians are increasingly recognizing that exercise in lung cancer simultaneously preserves everyday function—enabling patients to maintain functional capacity, independence, and the ability to manage daily activities at home. Prolonging the period of independent living is of substantial clinical relevance, and this Research Topic aims to drive a conceptual shift: lifestyle, and exercise in particular, is not merely a context for cancer care, but an active modifier of both disease trajectory and lived experience. A core theme emerging from this Research Topic is the interplay between body composition and lung cancer. Historically, weight loss, sarcopenia, and cachexia have represented a hallmark in lung cancer, especially for the advanced disease, given their worse impact on patients’ prognosis and treatment tolerance (1, 2). Nevertheless, recently, the landscape has been further complicated by the metabolic consequences of modern treatments. Targeted agents, in particular, while extending survival, are associated with unintended weight gain and remodeling of adipose tissue, raising concerns regarding long-term prognosis, metabolic comorbidities, and quality of life (3). This emerging pattern provides a strong rationale for integrating exercise-based interventions, specifically targeting body composition to promote favorable increases in muscle mass, thereby improving long-term weight control (4). Accordingly, one contribution to the present Research Topic presents a case in which structured exercise was successfully implemented to address lorlatinib-associated weight gain and unfavorable changes in body composition, providing proof of concept for further testing lifestyle interventions to address this treatment-related metabolic toxicity (Mancini et al.).

Beyond the metabolic alterations, lung cancer treatments, e.g., surgery, chemotherapy, and radiotherapy, are characterized by a progressive deterioration of symptom burden, quality of life, and functional capacity. Dyspnea, fatigue, cough, and reduced exercise tolerance frequently accumulate over time, contributing to a gradual decline in patients’ daily functioning and quality of life. These trajectories are not restricted to advanced disease, but can also occur in patients with an early stage of disease (Weijst et al.). Beyond their prognostic implications, these impairments often represent significant unmet needs for patients that are insufficiently addressed and may affect autonomy, daily activities, and treatment tolerance. Over the years, exercise has emerged as an intervention able to support patients from this perspective. Exercise has been demonstrated to be safe, even in complex clinical scenarios, e.g., elderly patients or those with bone metastases, and also capable of improving physical fitness, such as cardiorespiratory capacity, muscle strength, and body composition, thereby permitting the maintenance of patients’ independence. Moreover, the spectrum of exercise benefits extends to the aforementioned symptoms, enhancing patients’ quality of life throughout the lung cancer care pathway. Within this continuum, increasing attention has been directed toward prehabilitation, with investigations demonstrating that exercise can reduce postoperative complications, shorten hospital stays, and accelerate recovery.

Epidemiological studies have observed that physical activity, cardiorespiratory fitness, and muscle mass are associated with improved survival and reduced mortality and recurrence risks in lung cancer (5), suggesting that host functional reserve, modulated by exercise, may be clinically relevant for outcomes. At the biological level, exercise exerts systemic effects on metabolism, inflammation, and the tumor microenvironment, with implications for angiogenesis, the immune system, collectively influencing tumor-host dynamics (Mancini et al.). In this sense, immune modulation is particularly relevant in the era of immunotherapy: within this Research Topic, baseline host immune status, including higher lymphocyte counts, was associated with a major pathological response after neoadjuvant chemoimmunotherapy in resectable lung cancer (Wang et al.), reinforcing the concept that host biology can influence treatment efficacy.

Taken together, this evidence provides a strong rationale for hypothesizing that exercise may affect clinical outcomes in cancer. This will no longer be a purely theoretical speculation. The randomized CHALLENGE trial in colon cancer demonstrated that a structured exercise program after adjuvant therapy improved disease-free survival and suggested an overall survival benefit (6). Focusing on lung cancer, some studies are ongoing across different disease phases and therapeutic contexts (7). Among these, the NAVIGATE study is evaluating whether the integration of structured exercise within a comprehensive supportive care strategy can affect 1-year overall survival in patients with non-small cell lung cancer. In parallel, the STARLighT project is exploring the impact of combined exercise and nutritional interventions in two settings: first, in patients undergoing neoadjuvant chemoimmunotherapy, with pathological complete response and quality of life as co-primary endpoints; and second, in the adjuvant setting, with disease-free survival as the primary endpoint (7). Complementing these efforts, the EXHALE study showed that supervised exercise during chemotherapy is feasible and clinically beneficial even in advanced lung cancer, including both small cell and non-small cell disease, with improvements in muscle strength and patient-reported outcomes (8, 9) The growing body of evidence highlights emerging opportunities to build and strengthen coordinated, multidisciplinary research initiatives that advance exercise interventions across the lung cancer continuum. In this context, IREX-Lung has been established as a task force within the Nursing and Allied Health Professionals (NAHP) group of the International Association for the Study of Lung Cancer. The initiative serves as an international, multidisciplinary platform bringing together clinicians, exercise professionals, researchers, and patients to advance research on exercise and lifestyle interventions in lung cancer.

Collectively, the contributions in this Research Topic advance a paradigm in which exercise and lifestyle are integral, biologically active components of personalized lung cancer care. Viewed through this perspective, exercise as a lifestyle intervention should not be ancillary, but a dynamic, multifunctional lever capable of reshaping tumor-host interplay and enhancing the therapeutic landscape for patients with lung cancer.
